# The Hostel Peace Initiative: Rethinking Violence and Peace at the End of Apartheid

**DOI:** 10.1080/03057070.2024.2497704

**Published:** 2025-05-28

**Authors:** Franziska Rueedi

**Affiliations:** University of Zürich

**Keywords:** democratic transition, South Africa, political violence, hostels, migrant worker networks, traditional authorities, peacebuilding

## Abstract

In August 1992, one of the most remarkable peace initiatives was launched in South Africa when two warring migrant worker hostels began negotiating for a truce. The residents of the African National Congress-aligned Selby hostel and the Inkatha Freedom Party-dominated Jeppe hostel in central Johannesburg had been in open conflict for over a year. By February 1993, over 30 hostels had joined the Hostel Peace Initiative (HPI). Statistics show that violence in the greater Johannesburg region declined drastically during this period; the HPI, as this article argues, played an essential role in this decline. Amid intense political pressures and widespread violence, the HPI represented a grassroots effort by hostel residents to end the conflict. Based on archival research and oral history interviews, this article interrogates the significance of this initiative within the broader context of South Africa’s democratic transition. It explores the dynamics within and between hostels, mainly focusing on the roles of internal power structures, migrant networks and external political pressures. The article shows that* izinduna *(headmen) played a vital role in connecting the urban migrant worker hostels with the politics and concerns of the KwaZulu bantustan. Access to land and the future of the bantustan were key in mobilising traditional authorities in the hostels. The article aims to contribute to a fuller understanding of the significance of violence and peace during the transition to democracy in South Africa.

## ‘A vision of living together’

On 25 August 1992, Jacob Dlomo embarked on a journey that could have been his last. Dlomo, an employee at Absa Bank and a resident at Jeppe migrant worker hostel in central Johannesburg, was carrying a letter. When he arrived at the nearby Selby hostel on this late winter morning, he recalled, ‘I was scared to death’.^1^ The ‘blood on the walls and on the ground was still fresh from the fighting two days before’.^2^ The men at Selby hostel were allegedly stunned by Dlomo’s arrival but ‘delighted’ when they read the letter.^3^ It was a peace offering by some Jeppe hostel residents to the men at Selby hostel. ‘Until when will we kill one another?’ Dlomo allegedly asked the Selby men.^4^ ‘I have come to you to ask: “is there no other way we can live?”’^5^

The two hostels had been at war for nearly two years.^6^ Jeppe hostel, inhabited mainly by Zulu-speaking migrant workers, had gained notoriety as a stronghold of the Inkatha Freedom Party (IFP). At the same time, Selby had a large population of Xhosa-speaking residents who supported the African National Congress (ANC). However, as this article will demonstrate, party politics came to play a secondary role as different interests prevailed. The grassroots peace initiative was launched by Dlomo and other hostel residents who were, in their own words, ‘tired of being tools to perpetuate division and mistrust’.^7^ After consultations, older Selby residents appointed German Mlatsheni, a young man who had been a leader during the conflict, to represent them. At a press conference later, Mlatsheni emphasised the ‘sense of uncertainty’ the peace letter was met with but that they ‘honour[ed] the manner in which he [Jacob Dlomo] risked his life’.^8^

The men, working through the South African Council of Churches (SACC) and the ANC’s Peace Desk, sought Rev. Mvume Dandala’s assistance in mediating the peace process at Johannesburg’s Central Methodist Church.^9^ Dandala had learned from his father, a minister, how to resolve conflict in the villages and subsequently applied these insights. He recalled the atmosphere during an initial meeting and how the two warring groups reached out to one another:

They then said that what they want is to make peace. … I said, ‘ask them to share the words of peace’. … *Ukuthula kweNkosi makube nawe* [peace of the Lord be with you]. And the Xhosa would say *Uxolo lweNkosi malube nawe*. … I sat there for about 20 to 30 minutes. These chaps sharing the peace with one another. Because they knew one another before the conflict.^10^

The atmosphere was marked by shared grief over the loss of life encountered by both hostels: ‘[a]nd they would say, “and so and so, where is he?” And the other chap would say “*hayi* man, so and so, he died in this conflict”. And they would cry together. From both sides’.^11^ Importantly, the violence was not inflicted by distant strangers but was deeply intimate. Before violence shattered their friendships, the men from the two hostels had regularly crossed paths in sports competitions and cultural events. This shared history ultimately became the foundation for the peace initiative.

Agreements reached during their weekly meetings included leaving party politics at the door and not labelling hostels as ‘IFP’ or ‘ANC’ hostels (while keeping both parties informed about the initiative).^12^ Accusations and blame were prohibited, and participants avoided commenting on debates about hostel upgrades. These upgrades, including erecting fencing around hostels that were central to the conflict, were key recommendations of the Goldstone Commission.^13^ Many hostel residents fiercely rejected the Goldstone propositions, which had triggered renewed conflict in different areas.

A meeting on 21 November 1992 brought together 200 men from the Jeppe and Selby hostels and representatives from eight other hostels in Soweto and inner-city Johannesburg.^14^ ‘Almost every speaker at the meeting congratulated Mr. Domo [*sic*] for his courage’, a report noted.^15^ Four Johannesburg municipality officials and monitors from the United Nations Mission to South Africa (UNOMSA), churches and other international organisations attended the meeting. The hostel residents ‘pledged themselves to adopt a programme of preaching peace’, to resist the exploitation of ‘ethnic and tribal differences’,^16^ and to ‘call on their ancestor chiefs Shaka, Ncapayi, Saku and others to look kindly upon their children and help them to find unity and peace’.^17^ The participants collectively committed to fostering peace by organising mutual visits, actively encouraging others to abstain from violence, and establishing a steering committee to strengthen and expand the initiative.^18^ Feedback meetings were held in all the hostels involved.

Interestingly, a UN report observed that it ‘was obvious that the organizers did not want to use the peace structures, local or regional, or any political channels’.^19^ A statement of purpose confirmed the determination to keep the Hostel Peace Initiative (HPI) free from any outside influence, as participants feared the influence of different political interests.^20^ Conceived at the grassroots, the HPI would remain largely detached from national and international efforts at peacemaking.

As Dandala remembers, more hostels joined the peace initiative during the weeks that followed: ‘[t]hey [the Jeppe and Selby hostel residents] said, “let us have prayer meetings where hostels are going to be invited”. … We moved from hostel to hostel’.^21^ The expansion of the peace initiative was a key goal from the outset, as highlighted in the minutes of the 21 November meeting.^22^ Eventually, 32 hostels on the Reef signed a peace pledge.^23^ A Christmas prayer service on 19 December 1992 at Jeppe hostel was followed by an even larger service at Selby hostel in early February 1993, and a ‘candle of pain and hope’ was lit.^24^ To ‘seal the peace’, a cleansing ceremony was held in Alexandra, another township that had seen spiralling violence since March 1991; National Sorghum Breweries provided five cows for the ceremony.^25^ These rituals combined Christian symbolism with indigenous conflict resolution practices widely used across rural and urban African communities. At the heart of the process was the restoration of a moral community and expressing gratitude to the ancestors for their role in fostering peace.

Media reports in the aftermath of the service were met with criticism by the hostel residents. Dlomo expressed discontent with how hostel residents had been portrayed as ‘wild savages’ but given no credit for initiating peace.^26^ He emphasised that hostel residents, too, were human beings who had ‘learnt moral values from their parents, like everyone else’.^27^ His counterpart, Mlatsheni, further explained: ‘[t]here has been no victory for anyone. We make peace because of a higher value, a vision of living together in harmony. We do not make peace just because we are “weary of battle”, as one newspaper has suggested’.^28^ Contrary to media reports that seldom portrayed hostel residents as individuals with distinct beliefs and value systems, Dlomo, Mlatsheni and others emphasised their agency and shared humanity.

During the following months, the peace initiative grew. Another meeting on 6 March 1993 was attended by close to a thousand hostel residents from across the region. These peace efforts were remarkable, considering the viciousness of the violence that had torn through the region. Perhaps even more remarkable was the location of the 6 March meeting: it was held at Nancefield hostel, a notorious Soweto hostel where violence had been rife and allegations abounded that residents were involved in train attacks. The meeting resolved that each hostel would draw up a code of conduct that contained four key points: political tolerance, respect for cultural differences, refrain from ‘ethnic aggression’, and the promotion of ‘healthy relationships’ with township residents.^29^ In the second stage of the peace process, cultural programmes and sports events were to be held to bring hostel residents and their surrounding communities together.

The HPI was one of the most significant grassroots initiatives to foster peace in the Johannesburg region during the transition to democracy. It raises several important questions regarding the complex relationships between violence, resistance to violence, and peace and how they relate to party politics at the end of apartheid. First, what does the initiative reveal about power inside the hostels? What alliances emerged and how did they intersect with migrant networks? How did these alliances and networks relate to broader political concerns and material interests? Why, for example, did some of the hostels at the forefront of violence – like Mzimhlophe hostel in Soweto or Madala hostel in Alexandra – participate in the peace process, while the Vaal Triangle hostels did not? What other factors might we need to consider to understand these uneven responses to violence? Second, what is the agency of violence (and peace) at the local, regional and national levels? Third, what does the timing of this initiative reveal about the contingency and situatedness of violence? Could the HPI have enjoyed the same success a year earlier or a year later, or was there anything specific about the political and social context that enabled this initiative (or at least did not hinder it)? Fourth, what does the HPI reveal about indigenous practices and ideas of conflict resolution and peacebuilding? Furthermore, what do the initiative and broader responses to it reveal about the centrality of violence and peace during the transition to democracy? This article will not attempt to answer these questions in detail but will offer some reflections on under-researched aspects of transition violence.

## Archives of Violence

Collective violence in South Africa was at an all-time high in 1992 when the peace initiative was launched. In the first eight months of 1992, at least 2,843 people died countrywide and 3,840 were injured.^30^ In contrast to earlier forms of violence, the transition violence differed in both scope and forms: it was intimate, indiscriminate and often directed against entire communities. A special issue on political violence in southern Africa, published in the *Journal of Southern African Studies*, aimed to historicise and make sense of these new forms of escalating violence in South Africa and the region more broadly. William Beinart’s introduction to this special issue serves as a reminder to resist oversimplification and to shine a spotlight on a wide variety of dynamics and factors, including gender and socialisation, to name two.^31^ It remains one of the most insightful and influential analyses of the historiography of violence in southern Africa.

Since then, a growing literature has tackled the complexity and messiness of transition violence.^32^ The literature on hostels’ role in violence is particularly significant for this article. Lauren Segal’s seminal 1992 work, published at the peak of the violence, was groundbreaking in foregrounding the voices and experiences of hostel residents.^33^ Subsequent research has expanded on this scholarship by highlighting the diverse roots of the violence, ranging from socio-economic factors and material interests^34^ to the role of patriarchy and demographic shifts due to the abolition of influx control,^35^ competing ideas of nationalism^36^ and the marginalisation of rural migrant workers.^37^

This scholarship contributes to international literature exploring the multiple fault lines, patterns and motivations underlying collective violence. Stathis Kalyvas, in his widely cited study on civil wars, has emphasised the diverse allegiances and cleavages shaping violence in various case studies.^38^ He argues for the need to eschew rigid distinctions between private and political violence by exploring how personal grudges, revenge and private interests intersect with the ‘master cleavage’ at the national level.^39^ Consequently, as he has noted, the ‘loci of agency … are inherently multiple’.^40^ Leigh-Ann Fujii has demonstrated, in the case of the Rwandan genocide – one of the most horrific instances of intimate mass violence – the role that personal grievances played in motivating perpetrators to persecute and attack their Tutsi neighbours and kin.^41^ She shows that members of ethnic groups develop a variety of responses to violence and rarely act as a homogenous group. This fragmentation is compounded by the instability of categories. Since violence is dynamic, actors can change their ‘relations, perspectives, motives and identities’.^42^ The category of perpetrator (like any category) is therefore unstable: an actor may be a perpetrator of violence in one instant and evade or refuse it in a different context. Drawing on Kalyvas, Gary Kynoch has explored the complex reasons driving violence in South Africa.^43^ Both South African and international scholarship emphasise that the logic of macro-level conflict does not inevitably determine the reasons why people participate in violence and why they resist or reject it.^44^ Instead, it highlights the necessity to disaggregate ‘the masses’ to understand the motivations and roles of individual actors in driving the violence at the grassroots level and to understand their varied responses to violence in what Charles King has termed the ‘micropolitics of social violence’.^45^

Despite these insights, violence in South Africa has remained a slippery subject. Most works have focused on a particular region, and several lacunae are discernible.^46^ To date, no full-length monograph provides a comparative analysis of transition violence or a history of violence in South Africa.^47^ We know little about the networks that connected the epicentres of violent conflict during the 1990s – the Reef and KwaZulu-Natal – and rural authorities’ role in the Reef’s urban hostels. The role of the ‘third force’ remains shrouded in mystery: while we know about specific units and their nefarious deeds, many aspects remain underexplored.^48^ Finally, at the conceptual level, the relationships between violence and non-violence and the significance of peace initiatives during this period require more attention.^49^

Beinart’s article, as noted above, emphasised the importance of historicising and understanding violence in all its complexity. I argue that it is equally important to consider violence alongside resistance to violence, principled non-violence, and peace. To fully understand violence, one must also understand its absence.^50^ Asking what leads people to resist violence within a context of widespread conflict may help us understand issues of power, ideology, social norms and systems of belief differently. What factors might we need to consider to understand why some hostels did *not* participate in violence, resisted mobilisation into violence, and decided to settle their disputes and make peace? What different responses are discernible within hostels and what do they reveal about individual agency and motivations? What conditions enabled the HPI at this particular moment? If, as suggested above, violence requires agency, then resistance to violence must also require agency.

The relative lack of research on peace initiatives and the shifting relationships between violence and its absence are mirrored in archival collections. The transition era archive is vast and contains an almost dizzying number of documents: tens of thousands of pages chronicle escalating violence in the most severely affected regions. It is easy to get lost in this plethora of information, and patterns can be difficult to discern. Some observers have noted the uncanny occurrence of violence shortly after major political developments or after progress had been made in negotiations.^51^ However, in many other instances, a close look reveals largely contingent triggers and localised dynamics that led to a renewed escalation of violence.^52^ Silences in the archives are easy to overlook amid the abundance of information available. Notably, there is a lack of documentation on grassroots peace initiatives and quiescent areas. This gap is unsurprising, as key collections, like those from the Independent Board of Inquiry (IBIIR) and Peace Action, were focused on monitoring violence rather than peace.

This is particularly significant for understanding hostel dynamics. While much is known about KwaMadala hostel, infamous for the 1992 Boipatong massacre, far less is documented about hostels that remained quiescent, despite evidence that not all hostels on the Reef experienced violent conflict.^53^ Overall, with the few exceptions noted above, the records provide limited information on how hostels were structured and organised and how these structures related to migration networks during this period. Additionally, the complex interactions between factions of the IFP leadership, hostel *izinduna* (headmen), paramilitary units, vigilantes and criminal gangs remain unclear. This is partly due to a lack of records on the IFP, as few of its members testified at the Truth and Reconciliation Commission (TRC) and its archives remain largely inaccessible. Finally, other alliances – such as relationships between some hostels, the extreme right and the third force – remain challenging to explore as archival evidence is circumstantial and scattered.^54^

## Divided Hostels

It has been widely argued that the IFP launched an intensive recruitment campaign in hostels across the region after its launch in July 1990. Evidence suggests that among the first hostels to be mobilised were the Johannesburg and Soweto hostels, including Jeppe, Denver, City Deep, Jabulani, Nancefield, Mzimhlophe, Dube and Merafe.^55^ This recruitment preceded the outbreak of violence on 22 July 1990 in Sebokeng in the Vaal Triangle. On this day, a large ‘peace rally’, attended by thousands of hostel residents from across the region, as well as prominent IFP leaders, *amakhosi* (chiefs) and *izinduna*, led to widespread violence when attendees allegedly rampaged through Sebokeng township, leaving a trail of death and destruction in their path.^56^

During the following months, violence spread to the East Rand, the West Rand, Soweto and Alexandra hostels.^57^ Archival evidence points to different triggers that led areas to explode in violence. In the Kathorus region,^58^ conflict had simmered since 1989 over violent competition for taxi routes, predating the overtly political clashes of August 1990, as noted by Philip Bonner and Noor Nieftagodien.^59^ In Thokoza, it was rumoured that competition over a woman triggered the first cycle of violence in August 1990.^60^ The conflict rapidly spread and soon pitted residents of Phola Park, an informal settlement with a significant percentage of Xhosa-speaking migrant workers, against Zulu-speaking hostel residents, leading to the strengthening of ethnic solidarities. Some of the migrant workers from the Eastern Cape mobilised along their migrant networks, using traditional weapons and promoting a narrow form of nationalism.^61^ In the Vaal Triangle, on the other hand, the conflict largely saw Zulu-speaking hostel residents warring against urbanised township communities, with ethnicity playing no discernible role. The murder of a pregnant Sebokeng woman and the burning of IFP supporters’ houses allegedly heightened tensions before the 22 July rally.^62^ The expulsion of Zulu-speaking residents from Sebokeng hostel was a key aspect of the ensuing violence.^63^

The timing of violence also differed: while the East Rand township of Thokoza exploded in August 1990, and violence on trains in Soweto began to terrorise communities during the same month, Alexandra remained quiescent for several months and violence only flared in March 1991. There, a strong element in escalating violence was the crisis of the black local authorities. Shortly after calls to dissolve the Alexandra town council, Mayor Prince Mokoena allied with the local IFP.^64^ Local factors and dynamics, therefore, largely explain the different timing and uneven patterns of violence across the region.

Inside the hostels, the mobilisation into violence was also uneven and many were divided into peace factions and those advocating war. Many hostel residents joined only once the violence had flared and they felt under attack. A report, presumably written by the Congress of South African Trade Unions (COSATU) and focusing on the early violence in 1990, noted: ‘[i]t appears that certain Inkatha officials have been travelling from hostel to hostel, mobilizing hostel dwellers for war. People have been forced to attend meetings called by Inkatha, sometimes at gunpoint. This has resulted in clashes in the hostels’.^65^ The report further stated that: ‘[a]t the same time, Inkatha’s tactics have angered a lot of inmates, including their own constituency. In Jabulani, when Inkatha prepared the attack on the trains, inmates from KwaZulu told them that if they found that “the enemy” were ordinary people, they would attack Inkatha instead’.^66^

Evidence suggests that hostel residents mobilised along clan or regional networks to resist the violence during the early months. This resistance is significant as it complicates our understanding of hostel dynamics and highlights the diversity of responses. The few reports that do outline divisions between different factions explain them by place of origin: hostel residents from the Natal areas of Msinga, Ladysmith, Vryheid, Bergville and Estcourt were allegedly resisting the call to arms based on their clan networks and ‘en masse’.^67^ Migrant networks connecting hostels with rural areas played a vital yet understudied role in violent conflict.^68^

‘Warlords’ allegedly threatened to ‘speak to the chiefs from these areas because their people are refusing to participate in the fighting’.^69^ Conversely, those from ‘deep KwaZulu’, including Nkandla, Mahlabatini, Nongoma or Nquthu, allegedly advocated war.^70^ In some hostels, tensions reportedly simmered between residents from Natal and KwaZulu. Notably, attitudes toward violence also shifted, with migrants from Mahlabatini – who had been in the forefront of fighting – increasingly refusing to take up arms.^71^ Overall, the influence of peace factions eventually subsided as many residents opposed to violence left the hostels, making space for newcomers ready for violence. As an ANC press statement noted, ‘[s]ome men are leaving their jobs and going home. Those who have refused have been killed’.^72^ As this evidence makes clear, those opposed to the violence responded in different ways: some actively resisted from within the hostels, others avoided it by leaving urban areas, and still others, as the HPI demonstrates, actively promoted peace.

The IFP’s leadership also appeared to have been divided between peace factions and those advocating war: by October 1991 an ANC report, based on an IFP informant, noted that ‘divisions appear to be developing in IFP Transvaal over the nature of the IFP’s relationship with the hostel dwellers and the high incidence of violence associated with IFP rallies’.^73^ Sections of the IFP’s leadership opposed to the violence suggested that ‘the time had come to address the hostel dwellers personally’ and that ‘perhaps the best people to do this would be senior Zulu elders from Natal’.^74^ In contrast to widespread reports that painted Zulu-speaking hostel residents as a homogenous group readily available for violence, these archival records show the divisions inside the hostels and among the IFP’s leadership. During the early phase of the conflict, clan networks seem to have taken precedence over mobilisation based on political or ethnic lines. Religion, particularly the Nazareth Baptist Church founded by Isaiah Shembe, may have influenced hostel residents’ refusal to engage in violence.

As evidence suggests, rural concerns were often more important than ideology: *izinduna* and rural migrants were fighting for their way of life and the IFP best represented those interests.^75^ The influence of the KwaZulu government’s concerns and bantustan politics on the dynamics within hostels remains an underexplored area of research. A significant distinction lies in the differing status of traditional authorities in KwaZulu compared to Natal and how this intersected with issues of power, land access and other resources.^76^ The *amakhosi* in KwaZulu, acutely aware of threats to the bantustan system, were likely influenced by these pressures in their readiness to wage war. Threats to their rural base – and their reliance on the *amakhosi* – made migrant workers from these areas more willing to participate than those with fewer stakes in the future of the KwaZulu bantustan. Fear of losing access to land was thus only one of several factors that motivated rural migrants.

It is not surprising that regional or clan-based networks played a role in early mobilisation into, and resistance to, violence. The literature on early migrant worker culture has explored the significance of ‘homeboy’ networks and internal hierarchies and the centrality of migrancy to 20th-century South Africa.^77^ Many Zulu-speaking migrant workers retained strong ties to their rural base in KwaZulu and Natal, which they considered to be their real ‘home’ (*ekhaya*).^78^ Inside the hostels, these networks based on clans and regions persisted. Ari Sitas has explored the social organisation inside the East Rand hostels in detail. His research on the 1980s revealed that:

in the so-called Nguni hostels, most cultural formations (55 per cent of the cases) subsisted on an area or regional basis: people from a specific rural area (e.g. Xolo, or Phongola) interacted actively together after work. To a lesser extent such forms of association had an ethnic basis, e.g. Zulu or Xhosa or Shangaan (25 per cent). Finally only some associations (15 per cent) cut across ethnicity.^79^

These regional networks, although partly superseded by ethnic solidarity, nevertheless appear to have played a role in the early months of the conflict in late 1990 and early 1991.

## The Rural and the Urban: The *Izinduna*

I argue that the internal hierarchies within the hostels, along with how power was exercised and contested, played a crucial role in shaping patterns of violence throughout the region. Designed for social control, hostels operated within a strict hierarchy, structured along two lines of authority: official oversight by the (typically white) hostel manager and significant influence exerted by traditional authorities over their subjects. This was particularly the case for Zulu-speaking hostel residents. At the top of this hierarchy of traditional authorities was *Isilo* (the Zulu king), who exercised power through *amakhosi*, who in turn appointed *izinduna* to represent them in the rural areas and to provide a link between them and their subjects in the urban areas.^80^ The brother of an *inkosi* from Nquthu, who resided in the Kathorus region, recalled: ‘[f]rom time to time, … a delegation of *amakhosi* from different areas … come to [the Pretoria–Witwatersrand–Vereeniging [PWV] region] and meet their *izinduna* and address particular issues and then go back’.^81^ He acted as his brother’s representative in the urban areas:

He instructed me to be his eyes and ears here in [the PWV]. So whenever there are issues pertaining to *inkosi* … he would contact me and I would communicate with the people the message that he wants me to tell the people. … And even December time, we would have a big meeting … and talk to all our people.^82^

Evidence suggests that *izinduna* in the hostels originated without exception from the rural areas of KwaZulu and Natal.^83^ For larger groups of migrant workers from a particular area, two to three *izinduna* would be selected to represent the *inkosi*. Their primary responsibility was to resolve conflict, such as faction fights, and to ensure order and control.^84^ The interviewee from Nquthu recalled:

Everyone who stayed in those hostels during those days, they used to know who is who. Who stays where and where do you come from and who is your *inkosi.* Because remember there would be sometimes fights between men in the hostels. But then *izinduna* would intervene: if this one is fighting, if this one is from Nquthu for instance and is fighting somebody from Msinga … *izinduna* from both sides would have to intervene, to try and find a solution.^85^

Zulu-speaking hostel residents were, therefore, subject to forms of traditional authority that linked them to their rural homesteads under the authority of an *inkosi*. Significantly, the *izinduna* played a central role in allocating land on behalf of the *amakhosi* in the rural areas.^86^ Furthermore, as Gerhard Maré has pointed out, they controlled other resources. From scholarships to pensions, access to resources was under the control of the KwaZulu legislature and the *amakhosi*.^87^ Like black urban councillors in the townships, relationships were therefore steeped in patronage politics. This explains the power *izinduna* wielded in the hostels: disobeying an *induna*’s orders could have direct material consequences for the entire family in the rural areas. An ANC report from April 1991 highlights this: ‘[s]ome [hostel residents] have even been followed to their homes in Natal and Zululand, where they are further terrorised through denial of access to resources such as schools and pensions, and even threatened with death’.^88^

From mid 1990, IFP officials were mobilising *izinduna* across the region, yet the outbreak of violence accelerated this mobilisation. In the aftermath of the Sebokeng violence in July 1990, *izinduna* from across the region met in Johannesburg to discuss the way forward.^89^ Reports suggest that some hostels were under the power of ‘warlords’ aligned to the IFP.^90^ Some of the most detailed insights stem from Babylon Mgcina ka Xeketwane’s MA thesis on the Soweto hostels’ involvement in violence. He points out that in many Soweto hostels, where violence was rife, *izinduna* were ‘imposed from above by Inkatha hostel leadership’ and were expected to ‘enforce’ the decisions by the IFP’s Central Committee in Ulundi.^91^ Similar dynamics are discernible elsewhere: Bonner and Nieftagodien describe how the IFP seized Alexandra’s Madala and Nobuhle hostels in March 1991, expelling non-Zulu speakers and opponents after initial resistance, leaving behind a smaller, militant group of Zulu nationalists.^92^

However, the relationship between the IFP and *izinduna* in the hostels was more complex than it may appear. On 29 January 1991, the IFP and ANC issued a joint declaration to end the violence and to promote peace.^93^ Hostel residents’ refusal to engage in violence, as noted above, was partly motivated by the joint peace agreement between the IFP and ANC signed on this date.^94^ As an ANC memorandum details, a significant number of ‘warlords’ in the hostels were urging hostel residents to ignore these peace initiatives, claiming that ‘Buthelezi has “sold them out”’.^95^ At KwaMadala hostel, moderating influences appear to have been entirely absent and the hostel leadership were notorious for their belligerence and calls for violence. The ANC’s memorandum reveals that the IFP’s control over the hostels was less complete than some studies suggest, with direct contestation between the IFP Central Committee and certain regional hostel leaders. A report by the Ecumenical Monitoring Programme in South Africa (EMPSA) confirms this: ‘[t]here is a concern that “war talk” at a national level can destabilize sensitive peace moves at local levels, and “peace talk” at a national level has little impact at local level if it is not actively implemented’.^96^

In contrast to the *izinduna*, the *amakhosi* appear to have had limited control over the unfolding of violence in the urban areas. On 16 September 1990, less than two months after violence had erupted, Zulu King Goodwill Zwelithini and King Tutor Ndamase, leader of the Transkei, jointly called for peace. Traditional leaders from KwaZulu and the Transkei addressed hostel and township residents in the urban areas. In the East Rand, these efforts to end the violence were largely unsuccessful.^97^ An *induna* from Matubatuba who was representing his constituency in Thokoza recalls:

The emphasis was on peace, but while they were talking for peace the violence was escalating. And it is where the people of the ANC and IFP they entered in a meeting but the fighting was not for the political parties. We negotiated, but when the *amakhosi* went back, they started again. Because they used to meet as ANC and Inkatha. Because they knew each other, there was no fights among them and so when they finished negotiating everything is peaceful. But in the morning, there are more deaths.^98^

To understand why certain hostels became notorious for their role in the violence, it is crucial to examine who the *izinduna* were and whose interests they represented. As noted above, *izinduna* were divided between peace factions and those advocating war. One young hostel resident, who arrived in Katlehong from Newcastle at the height of the violence, recalls *izinduna* urging them to ‘*phansi imikhonto* [lay down your spears]’.^99^ Some of the *izinduna* attempted to restrain marchers. However, their orders were ignored: ‘[*n*]*oma izinduna zithi ziya khuza “Buya Zulu, buya Zulu” angithi nakhona zigijimela phambili kukhona ozothola* [even when the *izinduna* try to calm things down, saying “come back Zulu, come back Zulu”, they still rush forward because some would benefit [from the violence]]’.^100^ The quote highlights that in a context of violence, *izinduna* could lose control over the crowd, particularly when crowd members were driven by ulterior motives. Other *izinduna* were at the forefront of inciting violence: ‘[*k*]*ube khona lezi zinduna ezithile ezinamagazi ashisayo* [but there were those *izinduna* with boiling blood]’.^101^ These local and regional leaders were key actors in the violence, whose relationship with political leaders was complicated.

The increasing influx of young men destabilised the ‘gerontocratic rule’ in the hostels.^102^ The breakdown of the rural economy and the abolition of influx control in 1986 led new waves of young rural men to seek their fortunes in the industries in the PWV.^103^ Many struggled to find work, as jobs were scarce and competition fierce.^104^ As Segal has argued, older hostel residents often exerted a moderating influence over younger and more militant residents.^105^ Divisions within the hostels partly followed generational lines, with older men being ‘more ready to see themselves as mediators, voices of authority and rationality’ and younger men taking ‘the lead in organizing the violence’.^106^ These tensions also existed between older rural men and young urbanised members of the Inkatha Youth Brigade.^107^ In many instances, young men resumed leadership positions and directly challenged the authority of the older *izinduna*. As ka Xeketwane notes:

These youth occupied strategic positions within the hostel structures, breaking the monopoly of the Induna system. They either ignored or removed the prevailing hostel Indunas. Their leadership role was not questioned by their subjects because of the raging violence.^108^

In some instances, members of the Inkatha Youth Brigade took over the leadership of the hostel youth, though this trend cannot be generalised.^109^ Similarly, an ANC report noted the role of the Inkatha Youth Brigade in the violence.^110^ Rumours abounded that agents provocateurs had infiltrated both progressive organisations and the hostels to stir up violence.^111^ However, not all of these young men clashed with the older *izinduna*. Some, having earned respect for their leadership abilities, were appointed to leadership positions.^112^ Some young hostel residents were in open conflict with older *izinduna* over their criminal activities.^113^ Known as *izinkabi*, these young men were involved in looting, car hijackings and robberies. They came to play a leading role in the violence that plagued the urban townships around Johannesburg.^114^

Some hostels, like Denver hostel, gained notoriety for their involvement in gun-running. Through the networks of Renamo (Resistência Nacional Moçambicana), Koevoet and the South African Defence Force (SADF), large quantities of weapons were smuggled from Mozambique and northern Namibia to hostels in the greater Johannesburg region and KwaZulu and Natal.^115^ These networks played an important role in supplying hostel residents with weapons, as the regional commander of the Self-Protection Units (SPUs) emphasised: ‘[*i*]*zibhamu sasizithenga emaShanganeni ayefika nama-AK, ngenhlanhla sawathenga* [we used to buy guns from the Shangaans,^116^ who used to come with AK[-47]s, and luckily we bought them]’.^117^ In the East Rand, Nguni hostel in Vosloorus allegedly became an arms depot, where weapons supplied through Renamo networks were ‘lubricated and stored underground’.^118^

Key in this gun-running was the ‘third force’: sections of the security forces and their front companies. The Steyn Report exposed the nefarious activities of the military’s Directorate of Covert Collections (DCC), a branch of the military intelligence, along with the Special Forces (Spes Mag) and the Seventh Medical Battalion.^119^ Additionally, some members of the Civil Cooperation Bureau (CCB) – a hit-squad unit under the SADF that was officially disbanded in 1990 – were alleged to have unofficially reactivated secret structures.^120^ Under the code name ‘Operation Pastoor’, units of the security forces were implicated in the train massacres and the violence in the East Rand.^121^ The 1991 Inkathagate scandal further revealed the state’s secret funding of the IFP through the security branch. Evidence showed that Vlakplaas commander Eugene de Kock supplied the IFP’s Themba Khoza with weapons, with large quantities also delivered to the IFP in KwaZulu and Natal.^122^

The arming of the IFP’s paramilitary units was closely co-ordinated with their training. On 25 August 1993, the KwaZulu Legislative Assembly officially adopted the resolution to establish SPUs.^123^ Despite their name, these units became key instigators of violence on the Reef. Evidence suggests that the training of the IFP’s paramilitary units had been ongoing for several years prior to this: one camp, for example, was run by right-wingers at Entabeni outside Louis Trichardt.^124^ Under Operation Marion, the SADF trained IFP offensive paramilitary units in northern Namibia’s Caprivi Strip as early as the mid 1980s.^125^ Mlaba, the best-known SPU training camp, was established in 1993. Under IFP senator and former security branch policeman Philip Powell, between 5,000 and 7,000 IFP members were trained at Mlaba between October 1993 and March 1994.^126^ In its final report, the TRC noted that the SPU project, while containing elements of self-protection, was ‘intended to furnish the IFP with the military capacity’ to prevent the elections ‘by force’.^127^

This shadowy world stretched beyond southern Africa and formed part of a larger international network of organisations and individuals bent on halting the transfer of power and bolstering Buthelezi’s power base. British journalist Bruce Anderson, who was allegedly linked to the military intelligence, was central to facilitating gun-running networks.^128^ Malcolm Draper and Gerhard Maré have highlighted British zookeeper and honorary ‘white Zulu’ John Aspinall’s ties to the IFP and the International Freedom Foundation’s role in anti-communist politics.^129^ Aspinall’s circle helped fund the IFP, transferring about four million rand by 1994, viewing Buthelezi as a more viable alternative to the ANC.^130^ On the extreme right, international neo-Nazis were recruited to travel to South Africa, where they became involved in violent attacks.^131^

## 1992: Beyond the Massacre

On 14 September 1991, numerous political parties, trade unions and other entities signed the National Peace Accord. The signatories included the IFP but excluded extreme right-wing organisations – such as the Afrikaner Weerstandsbeweging (AWB) – and left-wing parties, including the Pan Africanist Congress and Azanian People’s Organisation (AZAPO). Liz Carmichael has recently shown that the Peace Accord provided a framework for holding different stakeholders accountable and for implementing measures to contain the violence.^132^ In some areas, the Local Dispute Resolution Committees (LDRCs, later Local Peace Committees) struggled to curb the violence. Mark Shaw makes this point in relation to Ratanda near Heidelberg: a lack of power and resources impeded its functioning and the Peace Accord was broadly seen as ‘imposed from the outside’.^133^ LDRCs were mainly successful in monitoring marches and rallies and liaising between different conflicting parties.^134^ In areas with hit-squad activities, violence flared despite the peace structures. The SPU commander of the Kathorus region recalls: ‘[*h*]*ayi, ayisebenzanga – babezidla nje imali* [no, that didn’t work – they were just chowing the money]’.^135^

The implementation of the Peace Accord in September 1991, therefore, had only a limited bearing on patterns of violence in the following months. In January 1992, the monthly death toll in the PWV was 40; but this rose dramatically during the first six months of 1992, peaking at 281 in June.^136^ This led the ANC to host an emergency summit on 22 April 1992. The IFP fiercely opposed the ANC’s calls for international monitors to be deployed to South Africa.^137^

As noted above, patterns of violence were heavily influenced by localised concerns; in many instances, attacks led to cycles of revenge that were difficult to stop. However, broader dynamics and interests during this period are equally important: the status of the negotiations and the IFP’s stake in them, the growing radicalisation of the extreme right-wing, the impact of the third force, as well as the concerns of the KwaZulu government and the *amakhosi* all had an impact on conflict on the ground. The first significant development was the launch of the Hostel Redevelopment Programme in October 1991, which faced strong resistance from hostel residents. Shortly after, in December 1991, the first Convention for a Democratic South Africa (CODESA 1) began, with additional talks resuming in May 1992. However, these subsequent negotiations ultimately reached a stalemate. Notably absent from CODESA were King Goodwill Zwelithini and the KwaZulu government, prompting Buthelezi to withdraw from the discussions. The period of increasing violence, therefore, corresponded with the IFP’s and the KwaZulu government’s opposition to CODESA.

On 17 June 1992, violence reached new heights when several hundred hostel residents descended from KwaMadala hostel in Vanderbijlpark and brutally murdered dozens of women, men and children in the township of Boipatong. The Boipatong massacre, more than any other during the transition period, sent shockwaves nationally and globally.^138^ While previous massacres had occurred in the PWV and Natal,^139^ Boipatong shifted power dynamics and halted negotiations. In response, the UN passed Resolution 765 on 16 July 1992, condemning the violence and urging peaceful talks. A month later, on 17 August 1992, Resolution 772 was unanimously adopted; it called for the deployment of international observers to ‘strengthen and reinforce the indigenous mechanisms set up under the Peace Accord’.^140^ As the resolution made clear, the peace and security of the entire region was at stake.

During the winter of 1992, violence in South Africa, therefore, became a global affair. After the Boipatong massacre, stopping the violence became a significant objective, not only at the regional and national levels, but also at the international level. In September 1992 the UN sent its observer mission, UNOMSA, to monitor the violence and to pave the way for peaceful, free elections. Under the auspices of the UN, the Organisation of African Unity (OAU), the European Union and the Commonwealth all sent observers to the country. This was not the first time that the violence of the 1990s had attracted international attention; in 1991 and 1992 the International Commission of Jurists had toured the country to assess the situation.^141^

Due to the breakdown of CODESA, South Africa was in a political lull, and it was during this period that violence began to decline. By September 1992 the monthly death toll for the PWV had decreased to 80 and by January 1993 it stood at 51.^142^ At the macro level, the decline in violence after the breakdown of CODESA is not surprising if some of the violence was instrumentally aimed at halting negotiations. After the Bisho massacre of 7 September 1992, hardliners in the ANC were marginalised and negotiations resumed. Interestingly, the signing of the Memorandum of Understanding on 26 September 1992 and Buthelezi’s opposition to it seemingly had limited impact on levels of violence in the PWV. However, a decline in violence in the PWV was matched with an increase in violence in Natal. Patterns of violence overall, therefore, did not necessarily correspond to political contestations at the national level.

The HPI was launched during this period of declining violence. While peace did not prevail across the entire region, the HPI nevertheless had a significant impact on containing threats of violence and fostering peace from below. Of key importance were weekly meetings held with representatives of the different hostels: during these meetings, risks were discussed, rumours dispelled and agreements reached.^143^ Members of the HPI would be dispatched to conflict areas to appeal to hostel residents and to mediate. Dandala recalls preventing violence in Soweto on 16 June 1993. Rumours of an attack on the IFP-aligned Jabulani hostel led residents to arm themselves. Alerted by Jacob Dlomo, a delegation from Selby and Jeppe hostels rushed over, with ANC-aligned Selby residents joining Jabulani to avoid its exclusive IFP association.^144^ Much of the HPI’s strength lay in the personal relationships participants cultivated, which became the foundation for their shared commitment to promoting peace throughout the region.

By January 1993, Johannesburg and Soweto had been largely pacified, as an HRC report noted: ‘[p]articularly encouraging is the Soweto sub-region, where, for the first time in over two-and-a-half years, not a single politically related death was recorded’.^145^ The East Rand townships, in contrast, remained volatile. Of the 51 deaths recorded for January 1993, 36 were in the East Rand, seven in the West Rand, two in Alexandra, six in the Vaal and none in Johannesburg and Soweto.^146^ In the aftermath of Chris Hani’s assassination in April 1993, violence in the East Rand entered a new and particularly vicious phase. In late May, out of 66 deaths in the PWV, 64 occurred in the East Rand.^147^ The death toll for July of that year was the second highest since the beginning of violence in 1990.^148^

To curb growing tensions among ANC supporters, the election date was set for 27 April 1994. For those who felt sidelined – the IFP, the KwaZulu government and the extreme right – the setting of the election date intensified underlying fears of a future political order that would marginalise them. After Hani’s assassination, police and army generals, including Lothar Neethling, Pieter Groenewald and Constand Viljoen, formed the ‘Committee of the Generals’ to resist the transfer of power.^149^ This led to the Afrikaner Volksfront (AVF), an alliance of over 20 far-right groups. The TRC reported that the AVF committed human rights violations and secretly collaborated with the security forces and the IFP.^150^ The extreme right gained momentum, with AWB membership surging.^151^ A key demand was self-determination: in May 1993, AWB leader Eugene Terre’Blanche threatened war unless granted the right to form an independent state.^152^

This demand and right-wing opposition to an ANC-led majority government contributed to the formation of the Freedom Alliance between homeland leaders, the KwaZulu government, the IFP, the Conservative Party and the AVF.^153^ In some areas like the Vaal Triangle and the East Rand, these relations between the right-wing and the IFP materialised on the ground: in the Vaal, the AWB and the IFP marched under a joint banner and the extreme right allegedly assisted hostel dwellers in both regions.^154^ In the Vaal, allegations also surfaced of a connection to the police.^155^ In the Kathorus area, violence became particularly acute when the townships of Thokoza and Katlehong were plunged into a state of civil war. Violence also escalated in the Vaal Triangle, although it took on different forms compared to Kathorus. However, it is misleading to read the transition solely through the lens of its worst-affected areas. Violence did abate in many areas, and the HPI played a key part in this.^156^

## Conclusion

In December 2018, I met with the former commander of the SPUs in the Kathorus region outside Thokoza’s notorious hostels. As we sat down, he produced a weathered piece of paper that had been folded and unfolded many times. Under the heading *Kathorus Simunye News*, the newspaper article from 1994 described how a few weeks after the first democratic elections in May 1994, the commander had called Peace Action to express his concern about the ongoing violence in the region.^157^ I was struck by this. He seemed to want to introduce himself as a man of peace, not as a man of war, as his position as SPU commander would suggest. He explained his motivation for the phone call as follows: ‘[*m*]*asikhulume ngoba ngasho mina ukuthi uma siqhubeka nokushiya abantu abaphatha izibhamu ngaphandle, i-peace angeke siyiyenze* [when we were busy talking, I said if we keep leaving the people who were holding guns outside the talks we won’t get peace]’.^158^ In this instance, those ‘holding the guns’ were the heavily armed members of ANC-aligned Self-Defense Units (SDUs) and the IFP-aligned SPUs in the hostels.

His comment points to an important aspect determining the success or failure of peace initiatives: they rarely succeed if they are implemented from above. Eventually, a first meeting with different parties to the conflict in Kathorus was held in late May 1994. A month later, on 25 June 1994, a historic meeting took place with the SDUs and SPUs, which had been at war for several years.^159^ Under the chairmanship of Rev. Mvume Dandala, selected for his experience in the HPI, the pacification and demilitarisation of the Kathorus region were negotiated. As a former SDU commander recalled, the talks were tense, with guns drawn on at least one occasion.^160^

Much like the HPI, peace talks in the Kathorus region underscore the key importance of grassroots efforts in peacebuilding. However, the dynamics in Kathorus also reveal that patterns of violence, while influenced by broader national political developments, cannot be fully explained by high-level politics alone. To understand why violence erupted in certain regions at specific times and subsided in others, it is essential to take seriously the local dynamics and the agency behind violence and peace, as this article has shown. Violence needs to be understood in relation to the absence of violence, be it non-violence on principle, resistance to violence or the quantitative quietening of it. While much research has been conducted to explain the outbreak of violence during the 1990s, little has been done to explain the peace initiatives. To some extent, this is understandable: escalating violence lent urgency to scholarly analyses providing a framework for it.

But these biases also reflect how violence and its opposite are conceptualised and understood: while violence needs to be explained, the absence of violence appears to be the natural state and, therefore, requires no explanation. In the case of South Africa during the early 1990s, the absence of violence within a context of widespread conflict *does* need to be explained. Looking at violence through the lens of peace highlights the different alliances and actors that were key in shaping its forms. It raises questions of timing and agency, as well as the relationships between national dynamics and localised concerns. Finally, it shows the absolute centrality of violence during the transition to democracy from the local, national and international perspective. Violence and struggles against it were therefore neither epiphenomenal nor aberrant expressions of conflict; instead, they were at the very core of contestations over the political and social order.


Franziska Rueedi *Senior Lecturer, Department of History, University of Zürich, Rämistrasse 64, 8001 Zürich, Switzerland; Research Associate, History Workshop, University of the Witwatersrand, Johannesburg, South Africa. Email: franziska.rueedi@uzh.ch*
https://orcid.org/0000-0002-4856-7570


## Footnotes

1 K. Kekana and A. Nolan, ‘Making Peace from Below’, *Challenge*, Johannesburg, April 1993, p. 3.

2 *Ibid*.

3 *Ibid*.

4 J. Carlsson, ‘With Church Help, Hostel Residents are Working to Stop Violence’, *Southern Africa Church News*, Johannesburg, 15 February 1993.

5 *Ibid*.

6 L. Carmichael, *Peacemaking and Peacebuilding in South Africa: The National Peace Accord, 1991–1994* (Woodbridge, James Currey, 2022), p. 434. For witness testimonies see, for example, University of the Witwatersrand, Historical Papers Research Archive (hereafter ZA HPRA), Records of Peace Action, AG2515, L2.2.1, ‘Selby Hostel Crisis’.

7 ‘Making Peace from Below’, p. 4.

8 *Ibid*., p. 3.

9 Rev. Mvume Dandala, interview with author, online via Zoom, 11 July 2024.

10 *Ibid*.

11 *Ibid*.

12 K. Cullinan, ‘Hostel Dwellers Make Peace’, *Work in Progress*, 91 (August/September 1993), p. 14.

13 The Goldstone Commission of Inquiry Regarding the Prevention of Public Violence and Intimidation was established in October 1991 under the Peace Accord to investigate political violence and intimidation.

14 Carmichael, *Peacemaking and Peacebuilding in South Africa*, p. 440; another source notes that the meeting brought together 67 hostel residents from nine hostels. See ZA HPRA, Records of the Ecumenical Monitoring Programme in South Africa (EMPSA), AG2466 F7, Memorandum: Johannesburg Hostel Residents’ Peace Initiative, written by Rev. Mvume Dandala.

15 United Nations Archives and Records Management Section (hereafter UN ARMS), UNOMSA, S-1938-0033-0005-00001, Meeting between (Zulu and Xhosa dominated) Hostels in Johannesburg, 21 November 1992, a report by UN monitors Hanna Hussen and Isaac Minta.

16 ZA HPRA, AG2466, F7, Memorandum: Johannesburg Hostel Residents’ Peace Initiative; UN ARMS, UNOMSA, S-1938-0033-0005-00001, Hostel Residents Point the Way Forward to Peace, by Rev. Mvume Dandala.

17 UN ARMS, UNOMSA, S-1938-0033-0005-00007, Report of the Nguni Peace Committee, 21 November 1992.

18 *Ibid*.

19 UN ARMS, UNOMSA, S-1938-0033-0005-00001, Meeting between (Zulu and Xhosa dominated) Hostels in Johannesburg.

20 The statement of purpose reads, ‘this initiative has emanated solely from the hostel residents and not from any organization, and it is on such a basis on which they wish to sustain the initiative’. See UN ARMS, UNOMSA, S-1938-0033-0005-00001, Hostel Residents Point the Way Forward to Peace.

21 Rev. Mvume Dandala, interview with author, online via Zoom, 11 July 2024.

22 UN ARMS, UNOMSA, S-1938-0033-0005-00007, Report of the Nguni Peace Committee.

23 For a list of hostels in the region, see A. Minnaar (ed.), *Communities in Isolation: Perspectives on Hostels in South Africa* (Pretoria, Human Sciences Research Council, 1993), Appendix F: List of Industrial & Mine Hostels in South Africa, pp. 257–62. Based on official statistics, the entire number of hostels owned by the Transvaal Provincial Authorities was 92, and not all of them were participants in violence (the Pretoria region, for example, remained quiescent). See UN ARMS, UNOMSA, S-1938-0033-0005-00001, Background Paper on Hostels Wits/Vaal. The list of the original 24 signatories of the peace pledge includes the following hostels: Alexandra, Antea, City Deep, Denver, Diepkloof, Dobsonville, Dube, George Goch, Jabulani, Jeppe, Joburg Central, Kagiso, Kelvin, Klipspruit, Kwesine, Mapetla, Mayi Mayi, Mzimhlophe, Nancefield, Orlando Power, Selby, Thokoza, van Beek and Vosloorus.

24 ‘Making Peace from Below’, p. 4.

25 Carmichael, *Peacemaking and Peacebuilding in South Africa*, p. 442.

26 ‘Making Peace from Below’, p. 4.

27 *Ibid*.

28 *Ibid*.

29 ZA HPRA, AG2466, F7, Hostel Dwellers Call for Respect of Each Other’s Rights, issued by the SACC Communications Department, 7 March 1993.

30 Human Rights Commission, ‘Summary Report on Repression for the Month of August 1992’, quoted in Nelson Mandela Centre of Memory (hereafter NMCM), Records of the Goldstone Commission, Box 36, File 141, Report of the Ecumenical Eminent Persons Group, which visited South Africa 22–27 September 1992.

31 W. Beinart, ‘Introduction: Political and Collective Violence in Southern African Historiography’, *Journal of Southern African Studies*, 18, 3 (1992), pp. 455–86.

32 For a recent overview, see L. Evans, ‘Violence in the South African Transition’, in T. Spear (ed.), *Oxford Research Encyclopedias: African History* (Oxford, Oxford University Press, 2024).

33 L. Segal, ‘The Human Face of Violence: Hostel Dwellers Speak’, *Journal of Southern African Studies*, 18, 1 (1992), pp. 190–231.

34 P. Bonner and N. Nieftagodien, ‘The Truth and Reconciliation Commission and the Pursuit of “Social Truth”: The Case of Kathorus’, in D. Posel and G. Simpson (eds), *Commissioning the Past: Understanding South Africa’s Truth and Reconciliation Commission* (Johannesburg, Wits University Press, 2002), pp. 173–203.

35 G.S. Elder, ‘Malevolent Traditions: Hostel Violence and the Procreational Geography of Apartheid’, *Journal of Southern African Studies*, 29, 4 (2003), pp. 921–35.

36 I. Chipkin, ‘Nationalism as Such: Violence during South Africa’s Political Transition’, *Public Culture*, 16, 2 (2004), pp. 315–35.

37 A. Sitas, ‘The New Tribalism: Hostels and Violence’, *Journal of Southern African Studies*, 22, 2 (1996), pp. 235–48.

38 S.N. Kalyvas, *The Logic of Violence in Civil War* (Cambridge, Cambridge University Press, 2006).

39 According to Kalyvas, the master cleavage can be political, religious or ethnic, for example.

40 Kalyvas, *The Logic of Violence*, p. 365.

41 L.A. Fujii, *Killing Neighbors: Webs of Violence in Rwanda* (Ithaca, Cornell University Press, 2011).

42 *Ibid*., p. 8.

43 G. Kynoch, *Township Violence and the End of Apartheid: War on the Reef* (Woodbridge, James Currey, 2018); G. Kynoch, ‘Reassessing Transition Violence: Voices from South Africa’s Township Wars, 1990–4’, *African Affairs*, 112, 447 (2013), pp. 283–303. See also F. Rueedi, ‘The Hostel Wars in Apartheid South Africa: Rumour, Violence and the Discourse of Victimhood’, *Social Identities*, 26, 6 (2020), pp. 756–73.

44 Beinart makes this point when he argues that ‘[v]iolence requires particular forms of agency’ in Beinart, ‘Introduction’, p. 473.

45 C. King, ‘Review Articles: The Micropolitics of Social Violence’, *World Politics*, 56, 3 (2004), pp. 431–55.

46 There is a significant literature on violence in different regions. Recent examples include Kynoch, *Township Violence*; M. Mchunu, *Violence and Solace: The Natal Civil War in Late-Apartheid South Africa* (Charlottesville, University of Virginia Press, 2021); D. Bonnin, ‘Space, Place and Identity: Political Violence in Mpumalanga Township, Kwazulu-Natal, 1987–1993’ (PhD thesis, University of the Witwatersrand, 2008); J.E. Kelly, *To Swim with Crocodiles: Land, Violence, and Belonging in South Africa, 1800–1996* (East Lansing, Michigan State University Press, 2018).

47 See J. Dlamini, S. Hassim, L.H. Phillips, C. Saunders, T. Simpson, J. Thumbran and D. Magaziner, ‘South Africa, 1994 + 30: A Conversation About History After Apartheid’, *The Journal of African History*, 65, 1 (2024), pp. 4–21.

48 See S. Ellis, ‘The Historical Significance of South Africa’s Third Force’, *Journal of Southern African Studies*, 24, 2 (1998), pp. 261–99; P. Pigou, ‘The Apartheid State and Violence: What has the Truth and Reconciliation Commission Found?’, *Politikon*, 28, 2 (2001), pp. 207–33; F. Rueedi, ‘The Boipatong Massacre of 1992: Traces, Silences, and Truth(s)’, *South African Historical Journal*, 74, 4 (2022), pp. 683–87.

49 The exceptions are Carmichael, *Peacemaking and Peacebuilding in South Africa* and M. Dandala, ‘A Perspective on Mission and Peacemaking’, *Missionalia*, 22, 1 (1994), pp. 5–12.

50 Kalyvas makes this argument too: see Kalyvas, *The Logic of Violence*, p. 48.

51 NMCM, Records of the Goldstone Commission, Box 36, File 141, EMPSA Report, ‘South Africa: A Threatened Future?’, July 1993; the argument is most forcefully made in a joint report by the Human Rights Commission and Community Agency fir Social Enquiry (CASE) to the International Commission of Jurists on 28 March 1992. See University of Fort Hare, National Heritage and Cultural Studies (hereafter NAHECS), Records of the African National Congress, UK/Ireland, 192/26, HRC and CASE, International Hearing Document 5. The Reef Violence: Tribal War or Total Strategy? Ka Xeketwane makes the same point in his thesis. See B.M. ka Xeketwane, ‘The Relation between Hostels and the Political Violence on the Reef from July 1990 to December 1993: A Case Study of Merafe and Meadowlands Hostels in Soweto’ (MA thesis, University of the Witwatersrand, 1995).

52 Bonner and Nieftagodien, ‘The Truth and Reconciliation Commission’; A. Minnaar, ‘East Rand Townships under Siege’, *Indicator South Africa*, 10, 4 (1993), pp. 67–70.

53 ZA HPRA, Records of the Independent Board of Inquiry into Informal Repression, AG2543, 2.2.3, Independent Board of Inquiry, ‘Fortresses of Fear: Hostels and Reef Violence’ (Johannesburg, Independent Board of Inquiry, n.d.); UN ARMS, UNOMSA, S-1938-0033-0005-00001, Hostels Associated with Violence.

54 Mark Shaw notes the support for the AWB in Heidelberg. See M. Shaw, ‘Hostels, Violence, and the Possibility of Permanent Peace: A Case Study of Ratanda Township near Heidelberg, Transvaal’, in A. Minnaar (ed.), *Communities in Isolation: Perspectives on Hostels in South Africa* (Pretoria, Human Sciences Research Council, 1993), pp. 156–68.

55 ZA HPRA, AG2543, A11, Inkatha Offensive in the Hostels, July 1990.

56 Rueedi, ‘The Hostel Wars’, pp. 759–62.

57 See, for example, ZA HPRA, AG2543, 2.2.5, IBIIR Report for the Period July 1990.

58 Kathorus refers to the Katlehong–Thokoza–Vosloorus region in the East Rand.

59 Bonner and Nieftagodien, ‘The Truth and Reconciliation Commission’ pp. 179–82.

60 Kynoch, *Township Violence*, p. 60.

61 DB, SDU central commander in Katlehong and migrant worker from Mount Frere, interview with author, Mount Frere, 12 February 2019. I have indicated place of origin in case of a strong rural connection.

62 BA, mntwana (prince) of the Zulu royal family and induna from Nongoma (KwaMadala hostel Vanderbijlpark), interview with author, Johannesburg, 5 March 2019.

63 Rueedi, ‘The Hostel Wars’.

64 NAHECS, UK/Ireland, 192/28, Memorandum from the Information Sub-Committee of the National Working Committee on Violence, 16 April 1991, p. 4; P. Bonner and N. Nieftagodien, *Alexandra: A History* (Johannesburg, Wits University Press, 2008), p. 361.

65 ZA HPRA, AG2543, A11, Inkatha Offensive in the Hostels, p. 2.

66 *Ibid*., p. 3.

67 NAHECS, UK/Ireland, 192/28, Memorandum from the Information Sub-Committee of the National Working Committee on Violence, p. 3.

68 Segal, ‘The Human Face of Violence’, p. 200.

69 NAHECS, UK/Ireland, 192/28, Memorandum from the Information Sub-Committee of the National Working Committee on Violence, p. 3.

70 *Ibid*.

71 *Ibid*.; ZA HPRA, AG2543, 2.2.3, Independent Board of Inquiry, ‘Fortresses of Fear: Hostels and Reef Violence’, p. 29.

72 NAHECS, UK/Ireland, 194/44, Press Statement of the African National Congress on Violence, issued by the Department of Information and Publicity, 27 April 1991.

73 NAHECS, Lusaka, 21/157, Developments in Inkatha Transvaal, 28 October 1991.

74 *Ibid*.

75 Segal has made this point: see Segal, ‘The Human Face of Violence’.

76 G. Maré, *Ethnicity and Politics in South Africa* (London, Zed Books, 1993).

77 P. Harries, *Work, Culture, and Identity: Migrant Laborers in Mozambique and South Africa, c. 1860–1910* (London, James Currey, 1994); D. Moodie and V. Ndatshe, *Going for Gold: Men, Mines, and Migration* (Oakland, University of California Press, 1994). For a recent overview, see P. Delius, ‘The Making and Changing of Migrant Workers’ Worlds (1800–2014)’, *African Studies*, 73, 3 (2014), pp. 313–22; W. Beinart, ‘A Century of Migrancy from Mpondoland’, *African Studies*, 73, 3 (2014), pp. 387–409.

78 Segal, ‘The Human Face of Violence’.

79 Sitas, ‘The New Tribalism’, p. 237.

80 BB, induna from Kranskop (Jabulani hostel Soweto), interview with author, Johannesburg, 19 February 2019.

81 BI, IFP official from Nquthu, interview with author, Germiston, 7 February 2019.

82 *Ibid*.

83 BB, induna from Kranskop (Jabulani hostel Soweto), interview with author, Johannesburg, 19 February 2019.

84 *Ibid*.

85 BI, IFP official from Nquthu, interview with author, Germiston, 7 February 2019.

86 BB, induna from Kranskop (Jabulani hostel Soweto), interview with author, Johannesburg, 19 February 2019.

87 Maré, *Ethnicity and Politics in South Africa*, p. 67.

88 NAHECS, UK/Ireland, 194/44, Press Statement of the African National Congress on Violence.

89 BA, mntwana (prince) of the Zulu royal family and induna from Nongoma (KwaMadala hostel Vanderbijlpark), interview with author, Johannesburg, 5 March 2019.

90 The term ‘warlord’ is frequently found in archival records. However, it is not particularly useful as it reveals little about the status or origins of these men.

91 Ka Xeketwane, ‘The Relation between Hostels and the Political Violence’, p. 117.

92 Bonner and Nieftagodien, *Alexandra*, p. 364.

93 NAHECS, OR Tambo, 76/814, Joint Declaration of the ANC and IFP.

94 NAHECS, UK/Ireland, 192/28, Memorandum from the Information Sub-Committee of the National Working Committee on Violence.

95 *Ibid*.

96 NMCM, Records of the Goldstone Commission, Box 36, File 141, EMPSA Report.

97 DB, SDU central commander in Katlehong and migrant worker from Mount Frere, interview with author, Mount Frere, 12 February 2019.

98 BB, induna from Matubatuba (hostel Thokoza), interview with author, Thokoza, 7 March 2019.

99 BH, SPU member and hostel resident from Newcastle (Kwesine hostel Katlehong), interview with author, Katlehong, 12 March 2019.

100 *Ibid*.

101 *Ibid*. The term ‘boiling blood’ refers to having a quick temper and proclivity for violence.

102 Segal, ‘The Human Face of Violence’, p. 202.

103 *Ibid*.

104 BH, SPU member and hostel resident from Newcastle (Kwesine hostel Katlehong), interview with author, Katlehong, 12 March 2019.

105 Segal, ‘The Human Face of Violence’, p. 202.

106 *Ibid*.

107 BD, member of the Inkatha Youth Brigade (Soweto), interview with author, Johannesburg, 21 February 2019.

108 Ka Xeketwane, ‘The Relation between Hostels and the Political Violence’, pp. 144–5.

109 *Ibid*., p. 134.

110 NAHECS, UK/Ireland, 192/28, Memorandum from the Information Sub-Committee of the National Working Committee on Violence, p.4.

111 BD, member of the Inkatha Youth Brigade (Soweto), interview with author, Johannesburg, 21 February 2019.

112 BF, induna from Matubatuba (Thokoza hostel), interview with author, Thokoza, 7 March 2019.

113 BH, SPU member and hostel resident from Newcastle (Kwesine hostel Katlehong), interview with author, Katlehong, 12 March 2019.

114 See T. Gibbs, ‘Inkatha’s Young Militants: Reconsidering Political Violence in South Africa’, *Africa*, 87, 2 (2017), pp. 362–86; P. Bonner and V. Ndima, ‘The Roots of Violence and Martial Zuluness on the East Rand’, in B. Carton, J. Laband and J. Sithole (eds), *Zulu Identities: Being Zulu, Past and Present* (Scottsville, UKZN Press, 2008), pp. 363–82.

115 South African History Archive (hereafter SAHA), Freedom of Information Programme Collection (FOIP), AL2878, A2.4.1.7.10.1.1, Highlights of Gun Running Report.

116 Shangaans here refers to Mozambicans.

117 BE, regional SPU commander and hostel resident from Mahlabatini (Madala hostel Thokoza), interview with author, Thokoza, 18 December 2018.

118 ‘Hostels Where “truckloads of arms were sold”‘, *Weekly Mail*, Johannesburg, 28 September 1990.

119 In 1992, F.W. de Klerk tasked General Pierre Steyn with heading an internal inquiry into allegations of third force activities in the SADF. See ZA HPRA, AG3288, Executive Summary of the Steyn Report, pp. 8–9.

120 *Ibid*., pp. 13–14. The CCB was implicated in a wide range of assassinations and plots to destabilise the negotiations.

121 *Ibid*., p. 9.

122 Eugene de Kock speaks about this in detail in his amnesty application. See SAHA, AL2878, A02.2.15.054, Truth and Reconciliation Commission (1997), Amnesty Application Eugene Alexander de Kock, Vol. 1., For a summary, see SAHA, AL2878, A2.4.1.6., E. de Kock Summary.

123 ZA HPRA, AG2543, A14.4, TRC Submissions/Reports: the KwaZulu Police.

124 *Ibid*. Other camps were established in different parts of KwaZulu and Natal. See ‘The SPU Training Project, report produced by the Investigative Task Unit (ITU)’. I am grateful to Howard Varney for making the report available.

125 Truth and Reconciliation Commission, ‘Truth and Reconciliation Commission of South Africa Report, Volume 5’, available at https://www.justice.gov.za/trc/report/finalreport/Volume5.pdf, retrieved 8 April 2025, p. 235.

126 ZA HPRA, AG2543, A14.4, TRC Submissions/Reports: the KwaZulu Police. See also H. Lynd, ‘The Peace Deal: The Formation of the Ingonyama Trust and the IFP Decision to Join South Africa’s 1994 Elections’, *South African Historical Journal*, 73, 2 (2021), p. 330.

127 Truth and Reconciliation Commission, ‘TRC Report, Volume 5’, pp. 235–36.

128 ZA HPRA, AG2543, 2.3.7, Independent Board of Inquiry: Right-Wing Directory, March 1996, p. 32; SAHA, AL2878, A2.4.1.7.10.3.2, Aanduidings van Moontlike Voortbestaan/Herlewing van die BSB, Aanhangsel A by OP Crusen Vorderingsverslag, 22 September 1992.

129 M. Draper and G. Maré, ‘Going In: The Garden of England’s Gaming Zoo Keeper and Zululand’, *Journal of Southern African Studies*, 29, 2 (2003), pp. 551–69.

130 *Ibid*., p. 555; Laurens van der Post was another key figure. See J.D.F. Jones, *Storyteller: The Many Lives of Laurens van der Post* (London, Simon & Schuster, 2001).

131 See ZA HPRA, AG2543, 2.3.7, Independent Board of Inquiry: Right-Wing Directory, p. 2.

132 Carmichael, *Peacemaking and Peacebuilding in South Africa.* See also David Storey, interview with author, online via Zoom, 9 October 2024.

133 Shaw, ‘Hostels, Violence’, p. 164.

134 David Storey, interview with author, online via Zoom, 9 October 2024.

135 BE, regional SPU commander and hostel resident from Mahlabatini (Madala hostel Thokoza), interview with author, Thokoza, 18 December 2018.

136 NMCM, Records of the Goldstone Commission, Box 36, File 139, Centre for Socio-Legal Studies, South African Conflict Monitor: January 1992; March 1992; NMCM, Records of the Goldstone Commission, Box 36, File 140, Centre for Socio-Legal Studies, South African Conflict Monitor: April 1992; August–November 1992.

137 University of KwaZulu-Natal, Alan Paton Centre and Struggle Archives, PC 126/1/7, Fifth KwaZulu Legislative Assembly 1992, V. 66, Box 7, p. 828.

138 For an analysis of the consequences of the Boipatong massacre, see J. Simpson, ‘Boipatong: The Politics of a Massacre and the South African Transition’ (MA thesis, University of Cape Town, 2009).

139 For a list of massacres, see NAHECS, UK/Ireland, 192/26, International Hearing Document 7: Special Briefing on Massacres. Human Rights Commission, 26 June 1992.

140 UN ARMS, S/RES/772(1992), Resolution 772 (1992) Adopted by the Security Council at its 3107th Meeting on 17 August 1992, available at https://digitallibrary.un.org/record/148221?In=en&v=pdf, retrieved 30 November 2024.

141 NAHECS, UK/Ireland, 194/96, Agenda for Peace. An Independent Survey of the Violence in South Africa by the International Commission of Jurists.

142 NAHECS, UK/Ireland, 192/26, Human Rights Research Council, Monthly Repression Report, January 1993.

143 Rev. Mvume Dandala, interview with author, online via Zoom, 18 July 2024.

144 *Ibid*.

145 NAHECS, UK/Ireland, 192/26, Human Rights Research Council, Monthly Repression Report, January 1993, p. 8.

146 *Ibid*.

147 Human Rights Commission, Weekly Repression Report for Week No. 21, from 19 May 1993 to 25 May 1993, available at https://africanactivist.msu.edu/recordFiles/210-849-22495/EB136_opt.pdf, retrieved 8 April 2025.

148 Minnaar, ‘East Rand Townships’, p. 68; ZA HPRA, A3399, A, Annexure D. Peace Desk. Report to the 4th Regional Conference, 29–31 October 1993, p. 1.

149 D. Beresford, ‘SA Generals Form Group to Oppose Black Rule’, *The Guardian*, London, 30 April 1993, available at https://africanactivist.msu.edu/recordFiles/210-849-22495/EB136_opt.pdf, retrieved 8 April 2025.

150 Truth and Reconciliation Commission, ‘TRC Report, Volume 5’, p. 236.

151 A. Guelke, ‘Political Violence and the South African Transition’, *Irish Studies in International Affairs*, 4 (1993), pp. 59–68. See also ZA HPRA, AG2543, 2.3.7, Independent Board of Inquiry: Right-Wing Directory.

152 R. Kennedy, ‘Right-Wing Rally Threatens Pretoria with Armed Revolt’, *The Times*, London, 31 May 1993, available at https://africanactivist.msu.edu/recordFiles/210-849-22495/EB136_opt.pdf, retrieved 8 April 2025.

153 J. Robinson, ‘Fragments of the Past: Homeland Politics and the South African Transition, 1990–2014’, *Journal of Southern African Studies*, 41, 5 (2015), pp. 953–67.

154 Rueedi, ‘The Boipatong Massacre’.

155 J. Battersby, ‘Evidence May Provide Key to Third Force in S. Africa’, *Christian Science Monitor*, Boston, 19 July 1993, available at https://www.csmonitor.com/1993/0719/19022.html, retrieved 3 April 2025.

156 UN ARMS, UNOMSA, S-1938-0033-0005-00001, Handwritten Notes: Hostel Residents Peace Meeting, 15 July 1993.

157 *Kathorus Simunye News*, courtesy of BE, SPU commander Kathorus region. The date of this article is not visible, but it was likely published in late May or June 1994.

158 BE, regional SPU commander and hostel resident from Mahlabatini (Madala hostel Thokoza), interview with author, Thokoza, 18 December 2018.

159 ZA HPRA, Records of Peace Action, AG2515, K10, Progress Report on SDU/SPU Integration Process – Kathorus, 16 August 1994.

160 CB, SDU commander (Thokoza), interview with author, Germiston, 14 December 2018.

